# Enterovirus A71 Phenotypes Causing Hand, Foot and Mouth Disease, Vietnam

**DOI:** 10.3201/eid2504.181367

**Published:** 2019-04

**Authors:** Hoang Minh Tu Van, Nguyen To Anh, Nguyen Thi Thu Hong, Le Nguyen Truc Nhu, Lam Anh Nguyet, Tran Tan Thanh, Nguyen Thi Han Ny, Vu Thi Ty Hang, Truong Huu Khanh, Ho Lu Viet, Do Chau Viet, Ha Manh Tuan, Nguyen Thanh Hung, Du Tuan Quy, Do Quang Ha, Phan Tu Qui, Le Nguyen Thanh Nhan, Guy Thwaites, Nguyen Van Vinh Chau, Louise Thwaites, H. Rogier van Doorn, Le Van Tan

**Affiliations:** Oxford University Clinical Research Unit, Ho Chi Minh City, Vietnam (H.M.T. Van, N.T. Anh, N.T.T. Hong, L.N.T. Nhu, L.A. Nguyet, T.T. Thanh, N.T.H. Ny, V.T.T. Hang, D.Q. Ha, G. Thwaites, L. Thwaites, H.R. van Doorn, L.V. Tan);; Children’s Hospital 2, Ho Chi Minh City (H.M.T. Van, H.L. Viet, D.C. Viet, H.M. Tuan);; Children’s Hospital 1, Ho Chi Minh City (T.H. Khanh, N.T. Hung, D.T. Quy, L.N.T. Nhan);; University of Oxford, Oxford, UK (P.T. Qui, G. Thwaites, L. Thwaites, H.R. van Doorn);; Hospital for Tropical Diseases, Ho Chi Minh City (N.V.V. Chau)

**Keywords:** Enterovirus A71, Picornaviruses, Vietnam, hand foot and mouth disease, viruses, phenotypes

## Abstract

We investigated enterovirus A71–associated hand, foot and mouth disease in Vietnam and found that, after replacing subgenogroup C4 in 2013, B5 remained the leading cause of this disease. In contrast with previous observations, this switch did not result in an explosive outbreak, and B5 evolution was driven by negative selection.

Enterovirus A71 (EV-A71)–associated hand, foot and mouth disease (HFMD) is a major problem in Asia. With >1 million cases reported across the region annually, HFMD is attributed to large numbers of hospitalized cases (*1*). In addition, EV-A71 often is associated with high case-fatality rates for those with severe HFMD disease (*2*,*3*).

EV-A71 outbreaks are usually associated with predominant subgenogroup switches (*4*). In Vietnam, a switch from subgenogroup C5 to C4 in 2011 coincided with an explosive outbreak, resulting 174,677 hospitalizations and 200 deaths (*2*). More recently, EV-A71 subgenogroup C4 was replaced by subgenogroup B5 in 2013, and subgenogroup C5 was sporadically detected (*5*–*7*). Yet, no comprehensive report about subgenogroup circulation, evolution, and associated clinical phenotypes of EV-A71 in Vietnam has been generated since 2013. We investigated these subgenogroups to inform development of intervention strategies and guide public health authorities in response to HFMD outbreaks.

## The Study

We used clinical samples derived from patients enrolled in a concurrent HFMD research program in southern Vietnam. In that program, patients with suspected HFMD of all severities are enrolled from 3 major referral hospitals in Ho Chi Minh City: Children’s Hospital 1, Children’s Hospital 2, and the Hospital for Tropical Diseases (*8*). This study was approved by the hospital institutional review boards (document no. 73/BB-BVND1, Children’s Hospital 1; document no. 03EI/BVND2, Children’s Hospital 2; and document no. 150/BVBND-KH, Hospital for Tropical Diseases) and the Oxford Tropical Research Ethics Committee (document no. OxTREC reference 1005-13).

During July 2013–July 2015, we enrolled 1,547 patients. We performed PCR and identified EV-A71 as the most common cause of HFMD (24.5%, 379). Of patients with EV-A71, 91 (24%) had grade 2b1 HFMD or above (Table 1), accounting for most (47.4%) of the 192 enrolled patients who had severe HFMD. 

**Table 1:** Demographics and clinical severities of enterovirus A71 in patients with hand, foot and mouth disease, Vietnam, July 2013–July 2015*

Characteristic	Total EV-A71 cases enrolled, n = 379	EV-A71 cases included for phylogenetic analysis, n = 146	Subgenogroup C4 cases, n = 10	Subgenogroup B5 cases, n = 136
Sex				
M	213 (56.2)	89 (61)	8 (80)	81 (59.6)
F	166 (43.8)	57 (39)	2 (20)	55 (40.4)
Median age, mo (range)	21.9 (14.3–32.1)	19.4 (13.2–30.8)	13.9 (15.5–23.5)	19.6 (13–31.2)
Discharge grade†				
1	168 (44.3)	78 (53.4)	0	78 (57.4)
2a	120 (31.7)	42 (28.8)	3 (30)	39 (28.7)
2b1	30 (7.9)	13 (8.9)	4 (40)	9 (6.6)
2b2	16 (4.2)	10 (6.8)	2 (20)	8 (5.9)
3	43 (11.3)	3 (2.1)	1 (10)	2 (1.5)
4	2 (0.5)	0	0	0
Death	0	0	0	0

*Values are no. (%) except as indicated. EV-A71, enterovirus A71. †Grade 1, mouth ulcers or vesicles or papules on hands, feet, or buttocks, with or without mild fever (temperature <39°C). Grade 2a, central nervous system involvement, (myoclonus reported by parents or caregivers only, temperature >39°C or ataxia). Grade 2b1, myoclonus observed by medical staff or history of myoclonus and lethargy or pulse >130 bpm. Grade 2b2, ataxia, nystagmus, limb weakness, cranial nerve palsies, persistent high fever, or pulse >150 bpm. Grade 3, autonomic dysfunction with sweating, hypertension, tachycardia, and tachypnea. Grade 4, additional cardiopulmonary compromise with pulmonary edema or shock syndrome.

We performed whole-genome sequencing on representatives of EV-A71–positive throat and rectal swab specimens with sufficient viral load (*9*). We obtained 146 EV-A71 complete genomes spanning the sampling period from July 2013 through April 2015. We removed 1 recombinant, a result of a recombination between 2 parental subgenogroup B5 strains, from our analysis (data not shown). Phylogenetically, 136 isolates belonged to the B5 subgenogroup and 10 belonged to the C4 subgenogroup (Appendix Figure 1). The C4 subgenogroup was sporadically detected from September 2014 onward (Table 2).

**Table 2:** Distribution of enterovirus A71 subgenogroups detected by month, Vietnam, July 2013–April 2015

Year and month	Subgenogroup		Total
B5	C4
2013			
Jul	5	0	5
Aug	6	0	6
Sep	9	0	9
Oct	10	0	10
Nov	15	0	15
Dec	3	0	3
2014			
Jan	2	0	2
Feb	0	0	0
Mar	3	0	3
Apr	6	0	6
May	2	0	2
Jun	5	0	5
Jul	4	0	4
Aug	6	0	6
Sep	5	2	7
Oct	16	4	20
Nov	16	1	17
Dec	11	1	12
2015			
Jan	3	0	3
Feb	0	0	0
Mar	6	2	8
Apr	3	0	3

**Figure 1:**
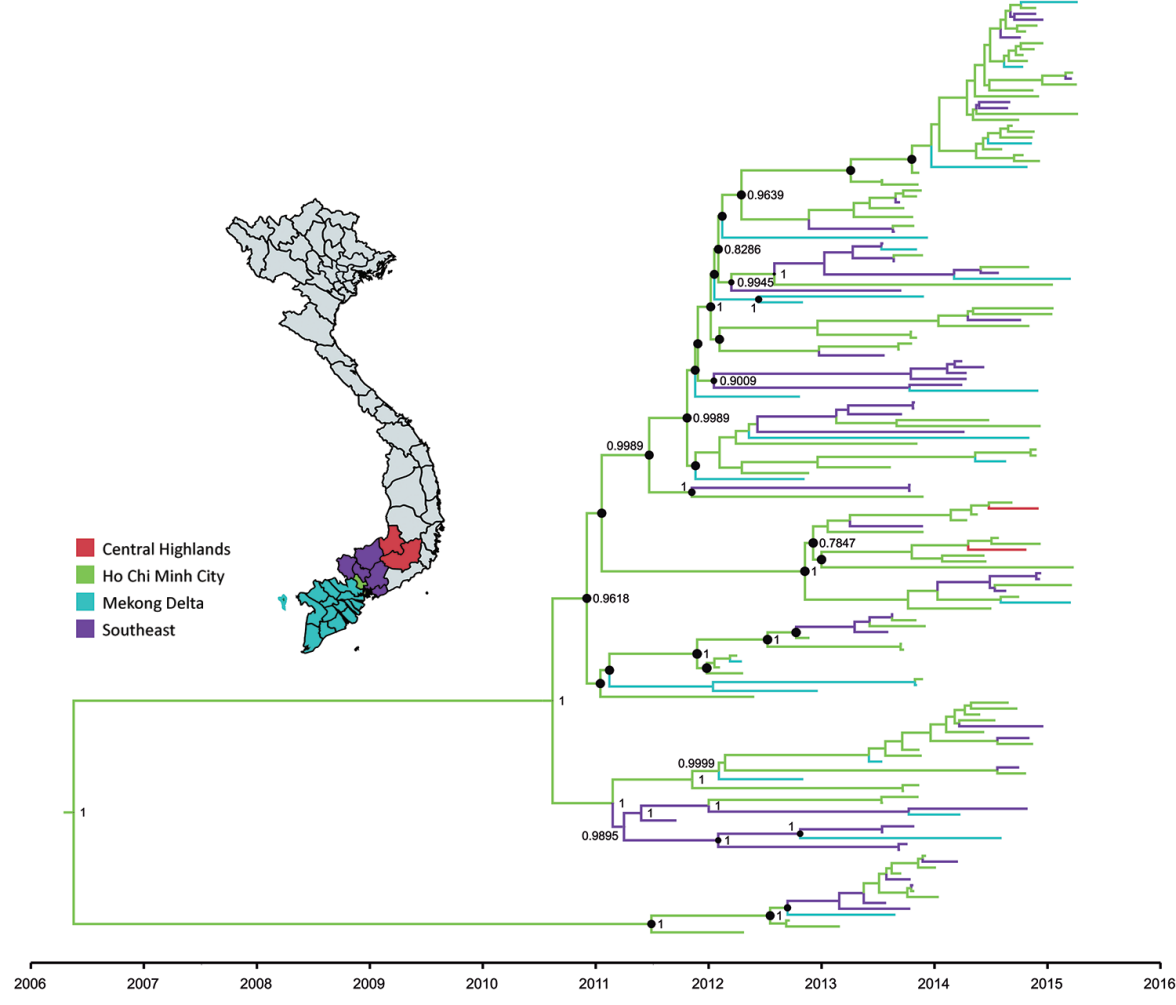
Maximum-clade credibility tree illustrating results of phylogeographic analysis of enterovirus A71 subgenogroup B5 coding sequences, Vietnam, July 2013–April 2015. Black circles indicate posterior probabilities ≥70% and state probabilities ≥70% at all nodes. Branch colors represent sampling locations from 5 discrete states in Vietnam (inset map; https://mapchart.net). Small sample sizes from individual provinces precluded phylogeographic analyses at a finer spatial scale. Except for Ho Chi Minh City, we grouped provinces in Vietnam from which we sampled viruses into discrete locations, including southeast (Ba Ria, Binh Duong, Binh Phuoc, Dong Nai, Tay Ninh, and Vung Tau Provinces), Mekong Delta (Can Tho, Dong Thap, Hau Giang, Kien Giang, Long An, and Tien Giang Provinces), and Central Highlands (Dac Nong and Lam Dong Provinces). We analyzed whole-genome sequence data using general time reversible plus gamma 4 nt substitution models suggested by IQ-TREE version 1.4.3 (http://www.iqtree.org). Viral protein 1–based analysis yielded similar results (Appendix Figure 2). Enterovirus A71 sequences generated in this study were submitted to GenBank under accession nos. MH_716248–6393 and KP_691643–66.

**Figure 2:**
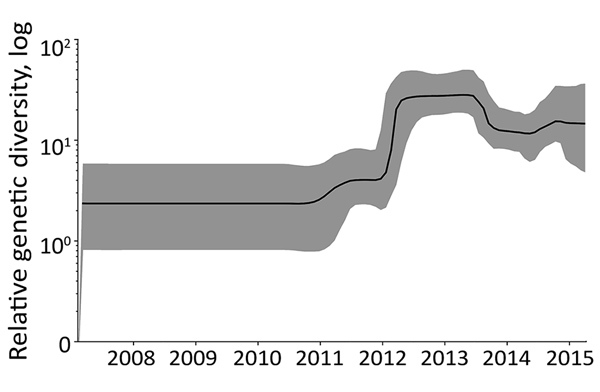
Complete coding sequence–based Bayesian skyline plot illustrating the relative genetic diversity of enterovirus A71 subgenogroup B5 in Vietnam over time. Black line indicates the mean; gray shading shows the upper and lower 95% highest posterior density values. Viral protein 1–based analysis yielded similar results (Appendix Figure 3f).

To unravel the evolutionary history of subgenogroup B5 in Vietnam, we used BEAST version 1.8.3 (*10*). The results of our analyses for main discrete geographic locations in Vietnam showed high fluidity within southern Vietnam, with Ho Chi Minh City being a likely source of viral circulation (Figure 1; Appendix Figure 2), supporting previously observed phylogeographic patterns of EV-A71 and other HFMD pathogens (*8*). Bayesian skyline analyses indicated that the relative genetic diversity of subgenogroup B5 increased sharply in 2012. This diversity was then maintained at a high level with slight fluctuations from 2013 to 2015, coinciding with a complete switch from subgenogroup C4 to B5 in 2013 (*6*) (Figure 2; Appendix Figure 3).

To estimate the rate of nonsynonymous (dN) and synonymous (dS) substitution, we used estimate selection for each codon, Z-test of selection, and Fisher exact test of selection methods available in MEGA5 (*11*). We estimated the nucleotide substitution rates among whole-genome sequences of EV-A71 subgenogroup B5 at 3.9 × 10^−3^ substitutions/site/year and for viral protein (VP) 1 sequences at 5.12 × 10^−3^ substitutions/site/year. Whereas no data exist for nucleotide substitution rates of EV-A71 whole-genome sequences, the substitution rate we estimated for VP1 sequences is slightly higher than that from previous reports (*6*,*12*).

Maximum-likelihood-based analysis revealed the estimates of mean dN:dS values were 0.0465 for VP1 and 0.0428 for complete coding regions, suggesting that EV-A71 subgenogroup B5 evolution was driven by strong negative selection, which supports previous reports (*6*,*12*). In contrast with findings from previous studies (*6*,*12*), our investigation for dN:dS ratios of individual codons did not reveal any sites, including VP1 residues 43 and 145, that underwent positive selection pressure. Because of the small number of subgenogroup C4 sequenced in our study, in-depth C4 phylogenetic analysis and comparison of associated clinical phenotypes between C4 and B5 were deemed uninformative. 

## Conclusions

Because others have extensively described the evolutionary history of EV-A71 on a global scale, including subgenogroup B5 (*5*,*6*), we focused our analysis on EV-A71 obtained from a comprehensive HFMD research program in Vietnam during July 2013–July 2015 (*8*) and the associated clinical phenotypes.

We showed that, after replacing subgenogroup C4 in 2013, subgenogroup B5 continued to circulate at a high level of endemicity and transmissibility, as reflected in our skyline plots (Figure 2; Appendix Figure 3) and phylogeographic patterns (Figure 1; Appendix Figures 1, 2), and was the major cause of HFMD in Vietnam, including cases with severe disease. However, compared with the 2011–2012 period, when subgenogroup C4 was circulating after replacing C5, the numbers of reported cases decreased during July 2013–July 2015, as did the proportion of fatalities attributed to HFMD in Vietnam (http://iris.wpro.who.int/handle/10665.1/14188). Of note, subgenogroup B5 exclusively circulates in the Asia-Pacific region and has been responsible for large HFMD outbreaks in Malaysia, Brunei, Taiwan (*13*,*14*), and, more recently, Thailand (*15*), whereas C4 has been circulating in China since 2008 and annually causes >1 million reported cases. Epidemiologically, subgenogroup switches often accompany large EV-A71–associated HFMD outbreaks (*4*,*6*). However, existing evidence fails to demonstrate the differences in terms of virulence and transmissibility between EV-A71 subgenogroups. Collectively, the underlying mechanism and factor determining pathogen emergence and the scale and severity of HFMD outbreaks, especially in specific localities, remains unknown, which might be a consequence of a complex interplay between cross-immunity, pathogen evolution, herd immunity, and public health responses.

The extent to which EV-A71 may adapt to in vitro cell culture systems remains unknown. We did not observe any specific amino acid residue that underwent positive selection in our analysis of subgenogroup B5 sequences from Vietnam. However, a recent study of subgenogroup B5 sequences from Vietnam generated by VP1 sequencing of B5 culture isolates recovered in RD and Vero cell lines worldwide showed that amino acid residues 43 and 145 of the VP1 protein are under positive selection (*6*). Because we obtained all EV-A71 genomes directly from clinical samples, our results could more accurately reflect the genetic diversity of EV-A71 in human populations, which might explain a slight difference in the estimated nucleotide substitution rate for VP1 sequences between our study and a recent report (*6*). Research to explore the potential biases introduced by the cell culture step on the observed genetic diversity of EV-A71 is urgently needed. Information obtained through such work could have profound implications for disease surveillance, which might also inform vaccine development and implementation.

Our study has some limitations. Because we based our surveillance only in southern Vietnam, the circulating viruses in the northern and central parts were not well represented. In addition, only EV-A71 samples with real-time PCR crossing point values ≤30 were subjected to sequencing, which might have resulted in an underestimation of EV-A71 diversity.

In summary, after replacing subgenogroup C4 in 2013, subgenogroup B5 EV-A71 continued to circulate at a high level of endemicity and transmissibility and remained the leading cause of HFMD in Vietnam, including cases with severe disease, during 2013–2015. However, this subgenogroup replacement event did not result in an explosive HFMD outbreak during the study period, and subgenogroup B5 evolution was entirely driven by negative selection. The underlying mechanisms and factors determining pathogen emergence, the scale and severity of outbreaks, and the extent to which EV-A71 may adapt to in vitro cell culture systems remain to be clarified.

Appendix Additional information on enterovirus A71 phenotypes causing hand, foot and mouth disease, Vietnam.
