# Supplementary material for: Enterovirus A71 Phenotypes Causing Hand, Foot and Mouth Disease, Vietnam
Source: Emerg Infect Dis. 2019 Apr;25(4):788–91. doi: 10.3201/eid2504.181367 (PMC6433038; doi:10.3201/eid2504.181367)
Supplement: Appendix — Additional information on enterovirus A71 phenotypes causing hand, foot and mouth disease, Vietnam. [file 18-1367-Techapp-s1.pdf]

Article DOI: https://doi.org/10.3201/eid2504.181367

Enterovirus A71 Phenotypes Causing Hand, Foot and Mouth Disease, Vietnam

Appendix

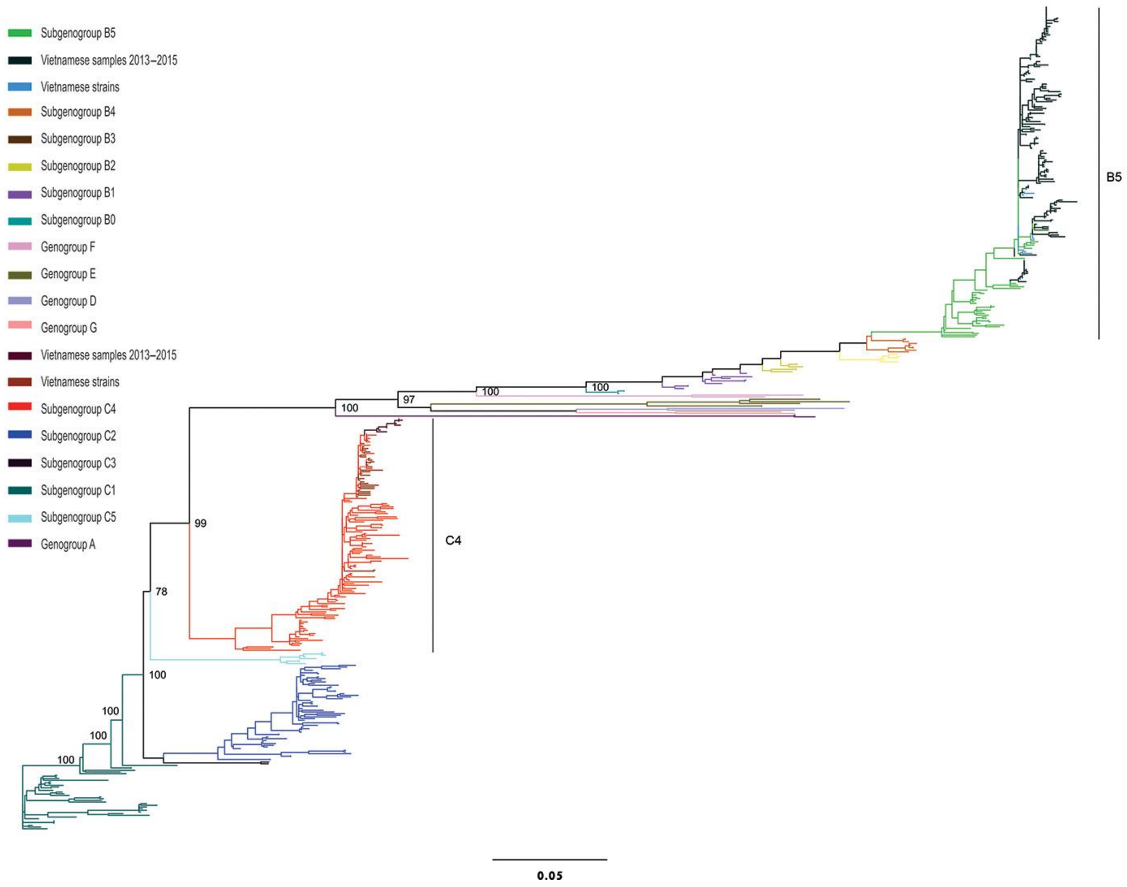

Appendix Figure 1. Maximum likelihood tree of representative VP1 sequences of all subgenogroups of enterovirus A71 (EV-A71), including sequences from this study. Scale bar indicates amino acid substitutions per site. Branch colors represent genogroups and subgenogroups. Numbers indicate bootstrap values. The sporadic detection of subgenogroup C4 in late 2014 and the tight clustering of those C4 sequences with previously reported C4 sequences from Vietnam suggest that, after causing the large outbreak in 2011–2012, subgenogroup C4 continued to circulate at a low level of transmission in Vietnam and cause asymptomatic infection that was not severe enough for the patients to come to the hospital.

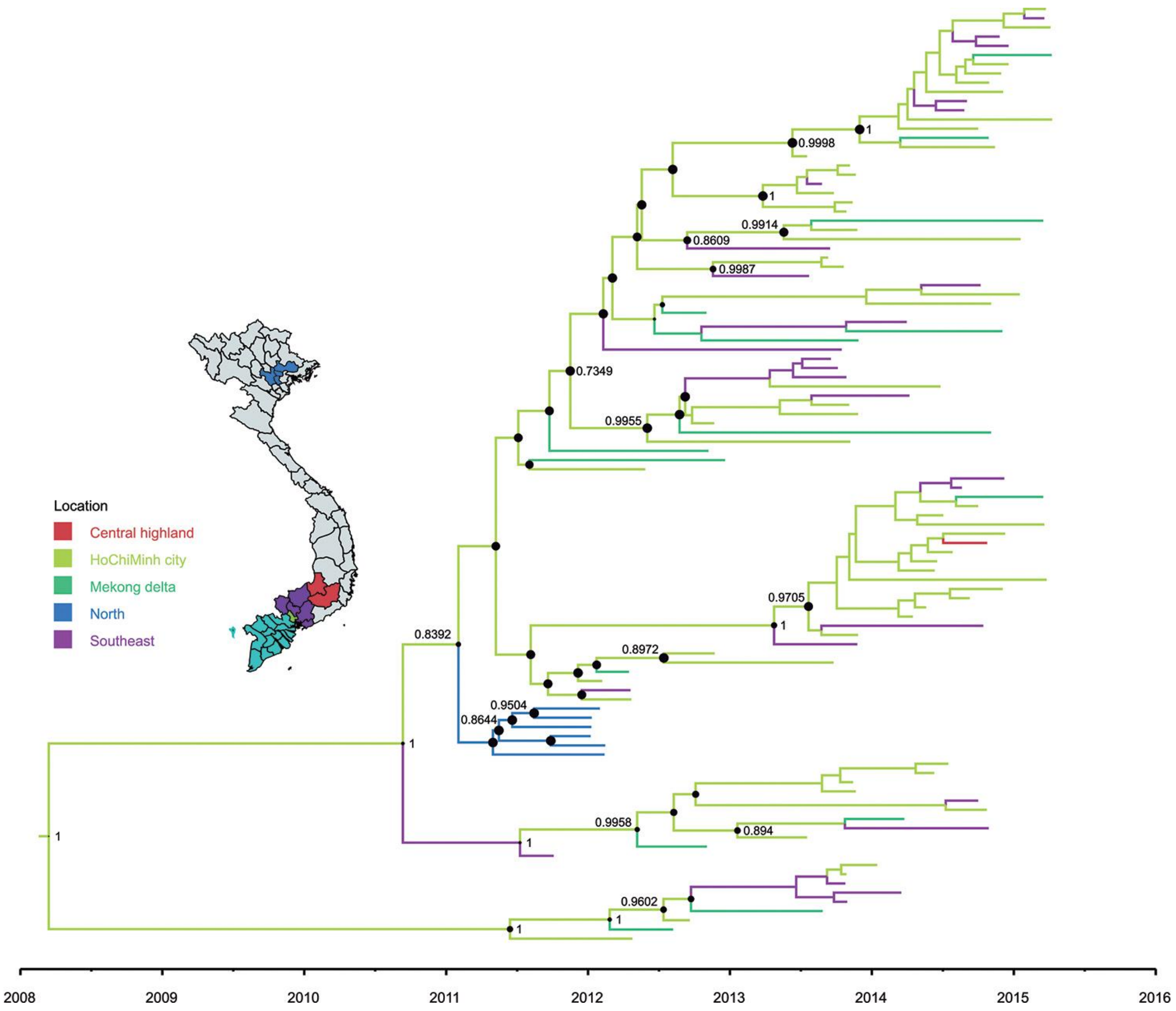

Appendix Figure 2. Maximum-clade credibility tree illustrating the result of phylogeographic analysis of enterovirus A71 subgenogroup B5 VP1 sequences. Branch colors indicate location sampling. Black circles indicate posterior probabilities ≥70% and state probabilities ≥70% at all nodes. We added the accompanying map (https://mapchart.net) to illustrate 5 discrete states in Vietnam. We analyzed VP1 sequence data using Tamura and Nei 1993 + gamma 4 nt substitution models suggested by IQ-TREE version 1.4.3 (http://www.iqtree.org).

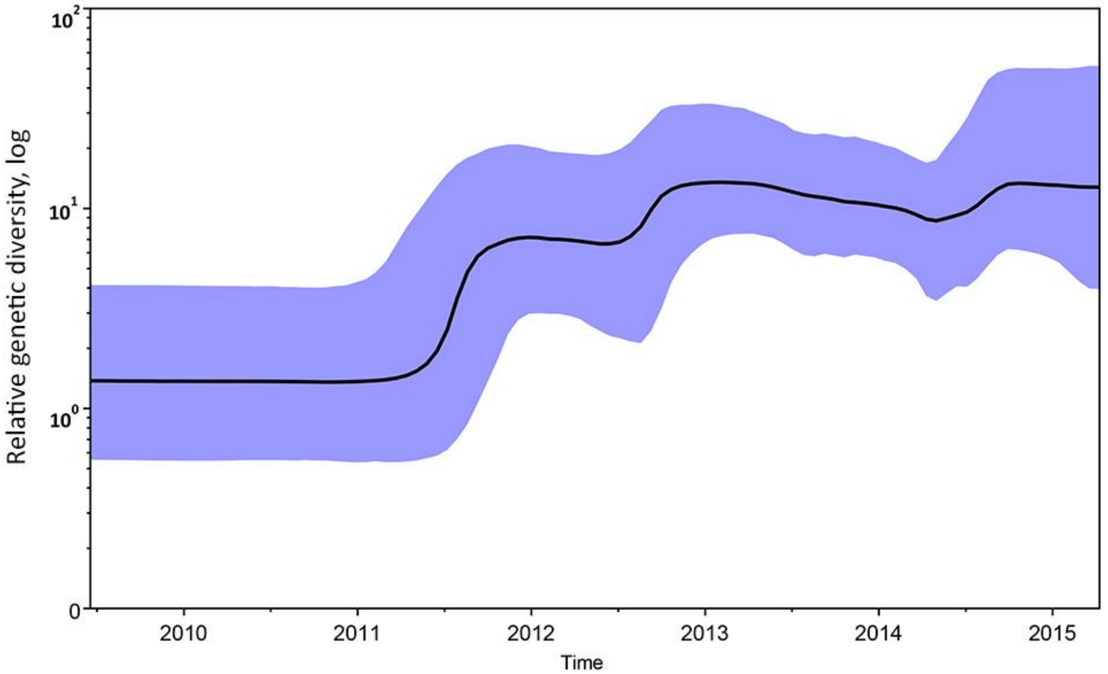

Appendix Figure 3. VP1 sequence-based Bayesian skyline plot illustrating the relative genetic diversity of enterovirus A71 subgenogroup B5 in Vietnam over time. The black line indicates the mean, the blue area shows the upper and lower 95% highest posterior density values.
